# An Overview of Autophagy in *Helicobacter pylori* Infection and Related Gastric Cancer

**DOI:** 10.3389/fcimb.2022.847716

**Published:** 2022-04-08

**Authors:** Yihan Yang, Xu Shu, Chuan Xie

**Affiliations:** Department of Gastroenterology, The First Affiliated Hospital of Nanchang University, Nanchang, China

**Keywords:** autophagy, *Helicobacter pylori*, gastric cancer, signaling pathways, therapy

## Abstract

*Helicobacter pylori (H. pylori)* infection is considered a class I carcinogen in the pathogenesis of gastric cancer. In recent years, the interaction relationship between *H. pylori* infection and autophagy has attracted increasing attention. Most investigators believe that the pathogenesis of gastric cancer is closely related to the formation of an autophagosome-mediated downstream signaling pathway by *H. pylori* infection-induced cells. Autophagy is involved in *H. pylori* infection and affects the occurrence and development of gastric cancer. In this paper, the possible mechanism by which *H. pylori* infection affects autophagy and the progression of related gastric cancer signaling pathways are reviewed.

## Introduction

Nowadays, the prevalence of gastric cancer has been increasing, which ranks the fifth among malignant tumors and the third most common cause of cancer death in the world (Tsai et al., 2017; Smyth et al., 2020). Although therapy for malignant tumors has significantly improved, the five-year survival rate of gastric cancer remains less than 30%. The relationship between *Helicobacter pylori (H. pylori)* infection and the prevalence of gastric cancer has been thoroughly identified. The World Health Organization (WHO) has listed *H. pylori* as a class 1 carcinogen, almost all gastric cancer cases are related to *H. pylori* (Ahn and Lee, 2015; Plummer et al., 2015; Schulz et al., 2016).

*H. pylori* was first recognized in 1983, which is a helical, highly mobile gram-negative pathogenic bacterium of 0.6 x 3.5micron size. For all that *H. pylori* is an extracellular pathogen, it can still cause persistent infection through residing and colonizing in the luminal surface of the gastric epithelium (Wroblewski et al., 2010). Numerous upper gastrointestinal disorders are associated with *H. pylori* infection, which is thought to infect over 50% of the worldwide population, which can cause gastritis, peptic ulcers, gastric cancer and so on (Blaser, 1987). *H. pylori* has such high pathogenicity, mainly depends on its various virulence factors, such as urease, vacuolar cytotoxin A (VacA), cytotoxin-associated-gene A (CagA), gamma-glutamyltranspeptidase (HpGGT), HP0175, high temperature requirement A (HtrA), and neutrophil-activating protein (NAP). In addition, some outer membrane proteins (OMPs), including outer inflammatory protein A (OipA), can also produce varying degrees of virulence (Pormohammad et al., 2018). In terms of the risk of gastric cancer, VacA and CagA are the most characteristic virulence factors, they both greatly promote the development of gastric cancer (Matos et al., 2013).

The specific mechanism of gastric cancer induced by *H. pylori* infection is not as clear as a bell. Previous studies have suggested that *H. pylori* can induce defective autophagy or inhibit autophagy, which enabling *H. pylori* to proliferate (Xie et al., 2020). It has been considered that VacA can disrupt lysosome activity, as well as CagA can enter gastric epithelial cells and induce interleukin-8 secretion (He et al., 2020). In addition, *H. pylori* can initiate abnormal activation of cellular signaling pathways, such as Nod1-NF-KB/MAPK-ERK/FOXO4 pathway, allowing cells to escape autophagy and leading to the occurrence of gastric cancer (Brandt et al., 2015; Eslami et al., 2019; Takahashi-Kanemitsu et al., 2020). Nowadays, autophagy have become the focus regarding the pathogenesis and targeted therapy in gastric cancer, This article reviews the effect of *H. pylori* infection on autophagy function and the role of autophagy in gastric cancer, so that can provide new targeted autophagy therapies and delay the progression of gastric cancer.

## Autophagy

### Mechanism of Autophagy

Autophagy is a common physiological process, it means to eat oneself, which is mainly used to provide energy and degrade some components in cells (Cuervo and Wong, 2014; Parzych and Klionsky, 2014). When cells are under stress conditions, such as starvation and hypoxia, the body phagocytoses diseased organelles or damaged cytoplasmic proteins to form autophagosome. Then it combining with lysosomes to form autolysosome, thereby maintaining the cellular homeostasis. This process can degrade and reuse degenerated cells, recycling their contents for subsequent use. On the one hand, autophagy can eliminate damaged cells when stimulated by adverse external factors, on the other hand, autophagy can promote tumor cell survival by preventing the accumulation of toxins or metabolites (Maes et al., 2013). Therefore, autophagy is a double-edged sword, and it has a two-way effect that can either promote or inhibit cell survival.

The mechanism of autophagy is simply the recognition of selective substrates by specific receptors, which are mainly divided into non-selective and selective isolation (Ryter et al., 2013; Lai et al., 2018). According to previous descriptions and summaries, the pathways of autophagy are mainly divided into the following three types, including chaperone‐mediated autophagy (CMA), micro-autophagy, and macro-autophagy (Mizushima and Levine, 2010; Galluzzi et al., 2017). CMA is a selective lysosomal pathway, the chaperone complex delivers the CMA substrate to the receptor protein on the lysosome membrane and assemble into a poly translocation complex. Besides, it can translocate cytoplasmic proteins into lysosomes, relying directly on recognition of targeted sequences by cytoplasmic HSC70, and then delivers cargo proteins to lysosomal-associated membrane protein2 (LAMP2). HSC70 is an essential prerequisite for CMA which can promote the classification of certain proteins in LAMP2 (Kaushik and Cuervo, 2012). Micro-autophagy mainly transfers cytoplasmic components to the lysosome, then the cholesterol-dependent vacuolar membrane directly invades into the lumen and compresses the newly formed vesicle finally. Simply speaking, micro-autophagy is a process that lysosome directly phagocytosis by intruding into lysosome membrane (Kaushik and Cuervo, 2012). Macro-autophagy is a process in which lysosomal fusion is involved in the breakdown of damaged organelles as well as the aggregation of proteins and pathogens. Importantly, macro-autophagy is the most reported autophagy subtype in *H. pylori* infection. Among up-regulating some survival promoting autophagy factors and down-regulating some death promoting apoptotic factors, macro-autophagy can alleviate the inflammatory response of *H. pylori* infection (Terebiznik et al., 2009; Yang and Chien, 2009).

### Autophagy-Related Genes

Shortly after the initial discovery of APG1-1, the autophagy research community identified nearly 70 autophagy-associated genes in different eukaryotes and named them autophagy-associated genes (ATGs) (Boneca et al., 2008). ATGs is involved in inducing membrane separation, cytoplasm phagocytosis, autophagy vesicles formation and fusion with lysosomes. More than 40 ATGs are related to the regulation of autophagy, the typical autophagy mechanism consists of more than 30 ATGs and was originally identified in yeast (Mizushima et al., 2011). ATGs constitute the core molecular mechanisms and functions of autophagy in several successive steps of the autophagy cascade to coordinate this process. ATG5 is the core components of autophagy related mechanisms, it is involved in elongation of the autophagosome (Yang and Klionsky, 2010; Reggiori et al., 2012). ATG7 is also critical to autophagy, the autophagy defect model is often be prepared by knocking out ATG7 in some experiments. Proteins in the ATG8 family are the first building blocks of the core autophagy mechanism and need to be characterized in molecular detail. ATG9 is the only complete membrane protein and plays a central role in mediating autophagosome formation. ATG11 is a scaffold protein required for selective autophagy and is also required for mitosis. The 62 amino acid regions of ATG11 are sufficient to support the autophagy function of ATG11 and ATG1 kinase activity. ATG16L1, a mammalian ATG16 homolog, is one of the gene clusters involved in autophagy, and mice lacking ATG16L showed impaired LC3 lipids. ATG19 bridges cargo proteins through the double membrane of ATG11 and ATG8 modified Cvt vesicles. In addition, ATG36 binds to ATG8 and the adapter ATG11, which associates selective autophagy type receptors with core autophagy mechanisms (Fujita et al., 2008).

Several studies have found that the levels of relevant ATGs expression are largely regulated in *H. pylori* infection, and inhibit autophagy function can promote the colonization of *H. pylori* in gastric epithelial cells (Odenbreit et al., 2000). The latest report found that a total of 28 ATGs were significantly down-regulated in *H. pylori*-infected AGS cells, and ATG16L and ATG5 were significantly lower in *H. pylori-*positive patients than in *H. pylori-*negative patients (Castaño-Rodríguez et al., 2015). To some certain extent, down-regulating some core autophagy genes can inhibit intracellular autophagy, which can further provide a good survival environment for *H. pylori* colonization.

## *H. pylori* Virulence Factors That Can Induce Autophagy in Gastritis Epithelium

*H. pylori* has many virulence factors, among which VacA, CagA, HpGGT and HP0175 can mainly induce autophagy in gastric mucosal epithelial cells. These virulence factors influence the autophagy flow by mediating relevant autophagy pathways, which in turn produce a series of different effects in cells (**Table 1**).

**Table 1:** The effect of *H. pylori* virulence factors that induce autophagy in gastritis epithelium.

Virulence Factors	Effect
VacA	Inhibits lysosome and autophagy killing Induces lysosomal damage Induces apoptosis Induces cellular damage Promotes the survival and colonization of *H. pylori* Promotes inflammation and the development of gastric cancer
CagA	Inhibits the formation of autolysomes Inhibits CagA-degraded autophagy Promotes inflammation and the development of gastric cancer Promotes the accumulation of CagA through VacA-mediated autophagy destruction
HpGGT	Disrupts lysosomal membrane integrity Reduced lysosomal cathepsin B activity Promote the internalization of *H. pylori* in gastric cells Promote the chronic gastric inflammation and gastric cancer
HP0175	Induces apoptosis Promotes autophagy through linking UPR

### VacA

VacA is a well-known virulence factor in *H. pylori*, which causes various cellular impaired functions in infected cells (Yamaoka, 2010). VacA toxin can also cause a series of changes in cells, such as vacuolization, disruption of lysosome function, and promoting immune regulation (Willhite et al., 2003). The pathogenicity of VacA is mainly determined by mosaic recombination between two main alleles (s1, s2, i1, i2, m1, m2) (Yamaoka, 2010). Strains with s1, m1 and i1 alleles produced more active toxins and were highly correlated with the occurrence of gastric cancer (Rhead et al., 2007).

Recent studies suggested that, the autophagy response process can be triggered by VacA, which can promote the formation of tyrosine phosphatase-related vacuoles as soon as activating VacA channels (Yahiro et al., 2012). Furthermore, VacA induces autophagy through endoplasmic reticulum stress, and inhibition of autophagy alleviates VacA-induced AGS cell death (Kroemer et al., 2010; Isomoto et al., 2010). VacA can induce autophagy when *H. pylori* temporarily infected gastric epithelial cells, which can degrade toxins, mitigate cell damage and further maintain cell homeostasis (Terebiznik et al., 2006; Terebiznik et al., 2009). But after long-term infection, it can destroy the autophagy pathway and further led to intracellular autophagy dysfunction (Ricci, 2016). When the cells were co-cultured with VacA for a long time, it can induce the collection of the autophagic substrate p62 and inhibit the expression of cathepsin D in autophagosome. P62 can connect with Rad51 subsequently, and then can increase the ubiquitination and degradation of Rad51, which suppress the function of repair damaged DNA (Xie et al., 2020). Thus, VacA mainly inhibits the autophagy of gastric mucosa epithelial cells in long-lasting *H. pylori* infection.

### CagA

CagA protein has structural diversity and strong immunogenicity, and is most related to *H. pylori* and gastric pathology (Odenbreit et al., 2000). The motifs of EPIYA in CagA strain can be divided into EPIYA-a, EPIYA-b, EPIYA-c and EPIYA-d. Western CagA strains usually include EPIYA-a, EPIYA-b and EPIYA-c sequences, while East Asian CagA strains only have EPIYA-a, EPIYA-b and a specific EPIYA-d, which can be partly explained by the highest incidence of gastric cancer in East Asian countries (Vianna et al., 2015; Kumar and Dhiman, 2018). Similar to VacA, CagA can induce a variety of cellular changes, including cytoplasmic vacuolation, endoplasmic reticulum stress, and mitochondrial dysfunction, and further lead to inflammation (Terebiznik et al., 2009). More and more evidence suggested that there may be a functional link between VacA and CagA virulence factors. Some articles have pointed out that there is a correlation between weak vacuolation and strong CagA phosphorylation, activing VacA is a key factor in CagA phosphorylation (Tsugawa et al., 2012).

CagA can be phosphorylated by tyrosine when it enters gastric epithelial cells, and tyrosine can be activated by a series of signaling factors, such as the PI3K/AKT/mTOR pathway signaling factors (Selbach et al., 2015; Tohidpour, 2016; Li et al., 2017). These can change the cytoskeleton of polyactin, causing an inflammatory response and suppressing autophagy (Mimuro et al., 2002; Li et al., 2017; Eslami et al., 2019). A new study suggests that CagA negatively regulates autophagy through the c-Met-PI3K/Akt-MTOR signaling pathway (Fan et al., 1996). In this study, compared with patients infected with CagA-negative *H. pylori* strain, SQSTM1 was significantly accumulated in the gastric mucosa of patients infected with the CagA-positive *H. pylori* strain. Besides, CagA-positive *H. pylori* significantly increased Akt phosphorylation levels at Ser473 as well as p-mTOR and P-S6 in AGS cells. In addition, CagA has been considered to increase the expression of miR-543, which can inhibit autophagy by combining with SIRT1, then further increase EMT and promote migration in gastric cancer (Semino-Mora et al., 2003; Suzuki et al., 2009). The expression of CagA is significantly higher in CD44 positive gastric cancer cells, mainly because CD44 positive gastric cancer stem cells can inhibit autophagy and further increase the expression level of CagA (Li et al., 2017). Even more important, CagA-positive strains can colonize in gastric mucosal epithelial tissues for a long time. It can upregulate inflammatory cytokines in *H. pylori* infected patients, and also can down-regulate related autophagy function. Therefore, the inhibition of autophagy by CagA promoted gastritis and thus initiated the occurrence of gastric cancer. Given the pluripotency of CagA, the interaction between CagA and autophagy regulation mechanism needs to be further studied.

### HpGGT

*H. pylori* gamma-glutamyl transpeptidase (HpGGT) is commonly found in all *H. pylori* strains, which plays a role in gastric mucosal epithelial cells (Chevalier et al., 1999). HpGGT, as an inhibitor of autophagy, can promote the internalization of bacteria in gastric cells, which can lead to chronic gastric inflammation and even gastric tumors. There has been suggested that HpGGT damages the autophagy flux by disrupting lysosomal membrane integrity in AGS cells, resulting in reduced lysosomal cathepsin B activity (Bravo et al., 2019). They considered a functional connection between HpGGT and VacA exists. In the early stage of *H. pylori* infection in AGS cells, VacA can induce autophagy, which can be seen through the transformation of the protein LC3 (microtubule-associated protein 1 light chain 3, MAP1LC3/LC3). In the late stage, HpGGT reduces cathepsin B activity and prevents lysosome degradation, which appears to inhibit autophagy. It should be noted that HpGGT inhibited autophagy after *H. pylori* infection in GES-1 cells, but which was not associated with cathepsin B. When *H. pylori* infects gastric epithelial cells, HpGGT inhibits autophagy and leads to genomic instability, which creates an environment conducive to carcinogenesis in turn. Thus, the exact mechanism by HpGGT regulates autophagy in various gastric epithelial cells remains to be determined, and how it promotes epigenetic changes in the progression of gastric cancer needs further study.

### HP0175

*H. pylori* contains a large number of secretory proteins, including HP0175, a peptide-based proline cis-trans isomerase (PPIase), that is highly recognized in serum and AGS cells of *H. pylori*-infected patients (Kim et al., 2002). Previous studies have shown that HP0175 induces apoptosis in gastric epithelial cells (Basak et al., 2005). Recent years, new studies have found that HP0175 can also mediate autophagy in gastric epithelial cells (Halder et al., 2015). HP0175 upregulates Beclin-1, ATG5, and UKL1 in AGS cells through transcription factors ATF4 and CHOP, in addition, it can induce LC3I to LC3II conversion in a PERK-dependent manner which depends on the unfolded protein response (UPR). This is the first time that HP0175 has been demonstrated to be associated with UPR and autophagy in *H. pylori*-infected gastric epithelial cells, and the in-depth mechanism of autophagy regulation still needs to further study.

**Figure 1:**
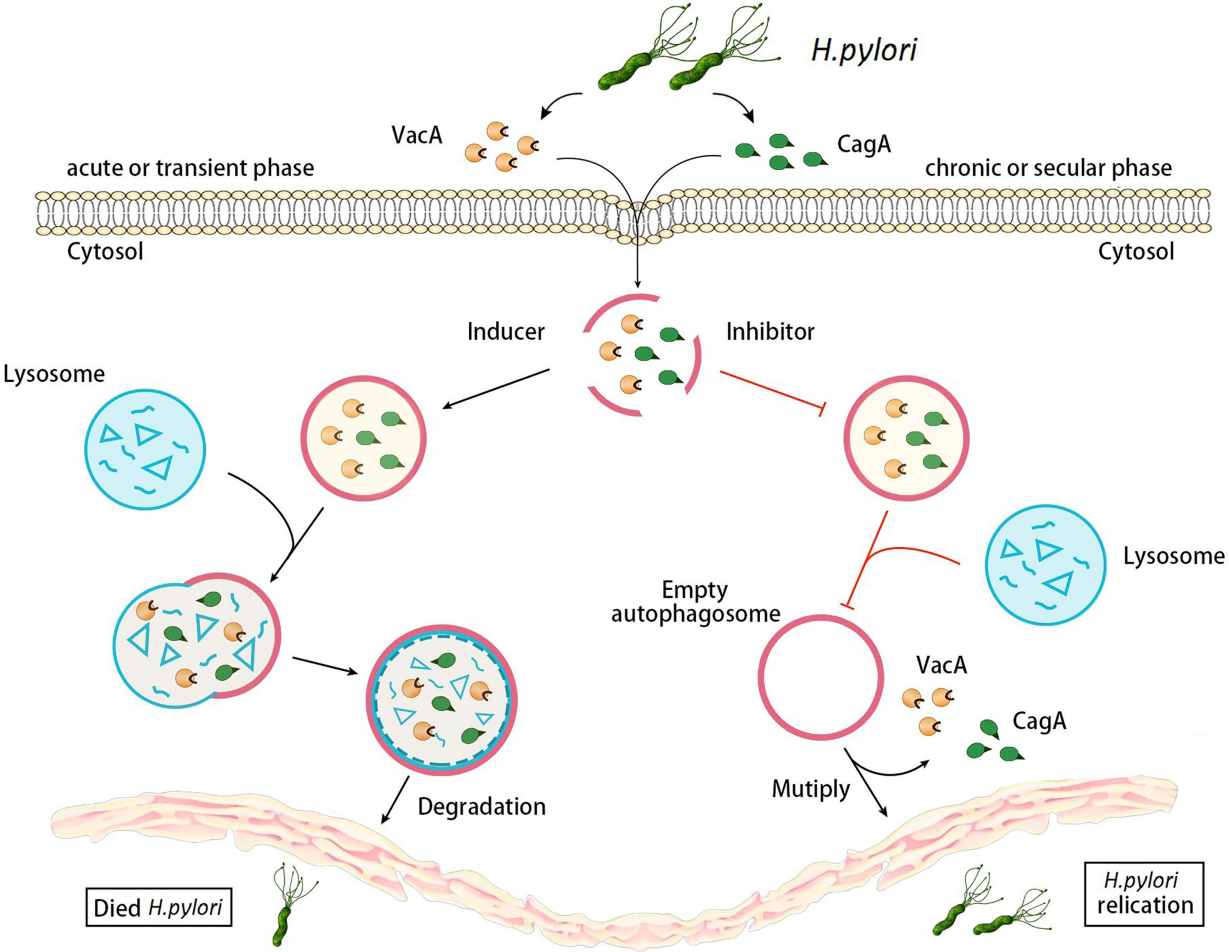
Changes of autophagy in gastric epithelial cells during different stages of *H. pylori* infection. During the acute stage of *H. pylori* infection, VacA and CagA secreted by *H. pylori* infection induce autophagy, and then autophagy degrades VacA and CagA. However, during chronic *H. pylori* infections, VacA and CagA inhibit autophagy, limiting the degradation of VacA and causing the accumulation of autophagy substrates. Moreover, autophagy destruction of VacA leads to the reducing degradation of CagA, which further enhances damage to host cells.

Autophagy is a dynamic process in gastric epithelial cells infected with *H. pylori*, it can respond to a range of disturbances in intracellular or extracellular homeostasis (Mizushima and Levine, 2010). In summary, autophagy protects gastric mucosal epithelial cells during the acute phase of *H. pylori* infection. VacA secreted by *H. pylori* induces autophagy, then autophagy degrades CagA and VacA, which reduce the damage in gastric mucosal epithelium. However, during chronic *H. pylori* infection, VacA destroys the autophagy pathway in gastric mucosal epithelial cells, limits the degradation of VacA, and results in the accumulation of autophagy substrates, causing severe DNA damage and accelerating the process of *H. pylori* infection-mediated gastric cancer pathogenesis (Zhang et al., 2019). Moreover, VacA damages autophagy function and then leads to the reduced degradation of CagA, which further enhances damage to host cells (Deen et al., 2015) (**Figure 1**).

## The Correlation Between *H. pylori* Infection and Core Autophagy Protein Markers

*H. pylori* can cause an increase in autophagosomes, it surrounded by a bilayer membrane is the characteristic of the formation in autophagosome (Allen et al., 2000; Allen, 2007). Intracellular *H. pylori* can induce autophagy by regulating autophagy-related genes, which further change a series of core autophagy protein markers.

### SQSTM1/p62

SQSTM1/p62, a ubiquitin-binding protein, which are both selective autophagy substrates and autophagy receptor proteins involved in autophagy (Puissant et al., 2012). It can deliver specific organelles and protein aggregates to autophagosomes for degradation. The accumulation of SQSTM1/p62 and ubiquitinated proteins can be used as indicators of autophagy inhibition, which serve as a receptor protein that can connect to the ubiquitinated structures of LC3B and misfolded proteins. The degradation of SQSTM1/p62 reflects the transformation of autophagy mechanism, and defective autophagy can result in the accumulation of SQSTM1/p62. Among them, E3 ubiquitin ligase TRIM32 can be used as a substrate for the autophagy receptor SQSTM1/p62, and targeted TRIM32 can degraded by the selective autophagy.

It has been previously reported that the protein levels of SQSTM1/p62 were significantly increased in *H. pylori-*infected gastric cells (Raju et al., 2012). Further animal models shown that SQSTM1/p62 levels are negatively correlated with Rad51 levels in *H. pylori-*infected gastric mucosa cells (Suarez et al., 2019). SQSTM1/p62 induces the ubiquitination of Rad51 mediated by *H. pylori* infection. The main mechanism is that *H. pylori* infection leading the nuclear translocation of SQSTM1/p62 through co-locating with Rad51, *H. pylori* infection can promote nuclear entry of SQSTM1/P62 and interaction with Rad51. Co-culturing AGS cells and GES-1 cells with *H. pylori*, when knockdown SQSTM1/p62 with the SQSTM1/p62 plasmid, Rad51 can be increased (Li et al., 2015).

In addition, clinical literature further found an increase of SQSTM1/p62 in gastric biopsies from patients infected with the virulent VacAs1m1 strain, compared with patients infected with the non-toxic strain VacAs2m2 (Tang et al., 2012; Lim et al., 2017). The accumulation of SQSTM1/p62 *in vivo* and *in vitro* biopsy samples from patients infected with the VacA-positive strain was mainly associated with impaired autophagy function. The expression of SQSTM1/p62 expression in gastric biopsies was also higher in patients infected with the CagA-positive strain than in patients infected with the CagA-negtive strain.

### MAP1LC3/LC3

Like SQSTM1/p62, MAP1LC3/LC3 is a core protein marker in autophagy, and MAP1LC3/LC3-associated phagocytosis (LAP) was found to play a key role in removing bacteria from some certain cells (Raju et al., 2012). LC3 can be divided into two subtypes, which are type I LC3 (LC3-I) and type II LC3 (LC3-II). LC3-I is the soluble form of LC3, and LC3-II is the membrane-bound form of LC3. In the formation of autophagosomes, LC3 undergoes multiple forms of transformation. Pro-LC3 is an untreated form of LC3, which is converted to LC3-I by hydrolyzed *via* ATG4. Conjugation of LC3 to lipids (LC3-II) requires the processing of LC3 precursors and LC3-I, which is mediated by ATG422, after that, ATG3 further converts LC3-I to LC3-II (Florey and Overholtzer, 2012; Deen et al., 2015; Li et al., 2017). In short, LC3-I conversion LC3-II (LC3-II/LC3-I) is a marker of autophagy induction. LC3 can bind directly to SQSTM1/p62, and LC3 binds to the LC3 interaction region (LIR) of p62 through the interaction between the n-terminal base residue of SQSTM1/p62 and the acid cluster on SQSTM1/p62. SQSTM1/p62 can interact directly with LC3 and even be degraded in autophagosomes (Kim et al., 2013; Mehta et al., 2014). The interaction between LC3 and SQSTM1/p62 is believed to be the mechanism that links the protein aggregates containing SQSTM1/p62 to the autophagy mechanism (Deen et al., 2013; Greenfield and Jones, 2013).

*H. pylori* infection triggered autophagy in gastric epithelial cells, which indicated by the presence of many GFP-LC3-positive structures clearly visible in the cytoplasm. Autophagy labeled LC3 dot structures co-located with *H. pylori* in *H. pylori*-infected AGS cells. Consistent with this finding, the expression of LC3-II and LC3 site formation were increased in AGS cells due to *H. pylori* infection. Furthermore, studies have found that VacA can induce vacuoles in gastric epithelial cells to act as the intracellular niche of *H. pylori*, then with the presence of LC3, it can further promote the fusion of lysosomal and the degradation of luminal content in vacuoles (Sanjuan et al., 2007; Barth et al., 2010; Glick et al., 2010; Florey et al., 2011; Martinez et al., 2011).

### Cathepsin D/CTSD

Cathepsin D(CTSD) is an aspartic acid protease in lysosomes, which is one of the main lysosomal proteases necessary for maintaining protein balance in cells. CTSD directly participates in autophagy and degradation of cell structures through reverse endocytosis and phagocytosis. Meanwhile, only CTSD can directly degrade the AGE transferred to the lysosome by endocytosis (Henault et al., 2012; Ma et al., 2012). It has been suggested that long-term exposure to VacA can lead to the formation of defective autophagosomes lacking CTSD and reduce their catalytic activity. It may be due to the fact that VacA neutralizes the acidic environment in the lysosomal lumen, which inhibits further maturation of proCTSD (Rijnboutt et al., 1992). Currently, it is believed that VacA can inhibit the fusion of autophagosomes with lysosomes in AGS, mainly because VacA changes the acidic environment of lysosomes, which can further cause the deficiency of CTSD, thus making the degradation function of autophagosomes unable to complete (Delbrück et al., 1994). Therefore, in line with the effect of VacA on the accumulation of SQSTM1/P62, the VacA-induced deficiency of CTSD can also promote the survival and replication of *H. pylori* in gastric epithelial cells^58.^

## The Autophagy Mediated Signaling Pathways in *H. pylori*-Associated Gastric Cancer

### The Nod1-NF-kB/MAPK-ERK/FOXO4 Pathway

NOD1, a member of nod-like receptor (NLR) family, is an intracellular immune receptor that senses peptidoglycan of Gram-negative bacteria (Allen, 2007). NOD1-specific single nucleotide polymorphisms (SNPs) have been associated with *H. pylori* infection and gastric cancer (Puissant et al., 2012). It was found that co-culture with *H. pylori* can up-regulate the mRNA and protein levels of NOD1-RIP2-NF -κB in gastric cancer cells, and also found that NOD1-RIP2-NF -κB pathway can activate autophagy. NOD1 receptor can detect intracellular *H. pylori* and recruit ATG16L1 to the entry site of bacteria, then it can regulate lysosomal and activate the NF -κB signaling pathway at the same time, which can cause the occurrence of inflammation (Blaser, 1987). In addition, long-term stimulation of *H. pylori* lysate promoted the phosphorylation of ERK, then it inhibited the levels of FOXO4 and its downstream genes, such as BNIP3, BCL-6, and ATG12, which indicated that the FOXO pathway can regulate cell autophagy. Therefore, *H. pylori* can inhibit autophagy and apoptosis through the Nod1-NF-kB/MAPK-ERK/FOXO4 signaling pathway.

### The Wnt/β-catenin Pathway

The Wnt/β-catenin signaling pathway is involved in multiple physiological processes, such as growth and development, cell regeneration, and organ formation. The abnormal proliferation of gastric cancer cells is related to their abnormal activation (Wang et al., 2019). When Wnt signal ligand binds to the cell membrane surface receptor protein frizzled, it activates proteins scattered throughout the cell, thereby activating Wnt signaling. These scattered proteins inhibit the activity of β-catenin complex, thereby stabilizing the free β-catenin in the cell. When β-catenin turns into the nucleus, it attaches to the T cell factor (TCF)/lymphoid enhancing factor (LEF) transcription factor family to promote downstream target gene (e.g., c-myc, cyclin D1) transcription, and abnormal activation of target genes promotes the proliferation and growth of tumor cells, inducing gastric cancer pathogenesis.

Recent research has revealed that, in the autophagy regulation process, the Wnt/β-catenin signaling pathway plays a crucial role in tumor cells (Yong et al., 2016). After *H. pylori* infection, autophagy is reduced by VacA, the CagA secretion system is activated, and β-catenin is accumulated in cells. This accumulation activates the Wnt signaling pathway and accelerates the activation of downstream genes, such as c-myc and cyclin D1. Therefore, this transcriptional activity leads to the onset of gastric cancer.

### The PI3K/Akt/mTOR Pathway

**Figure 2:**
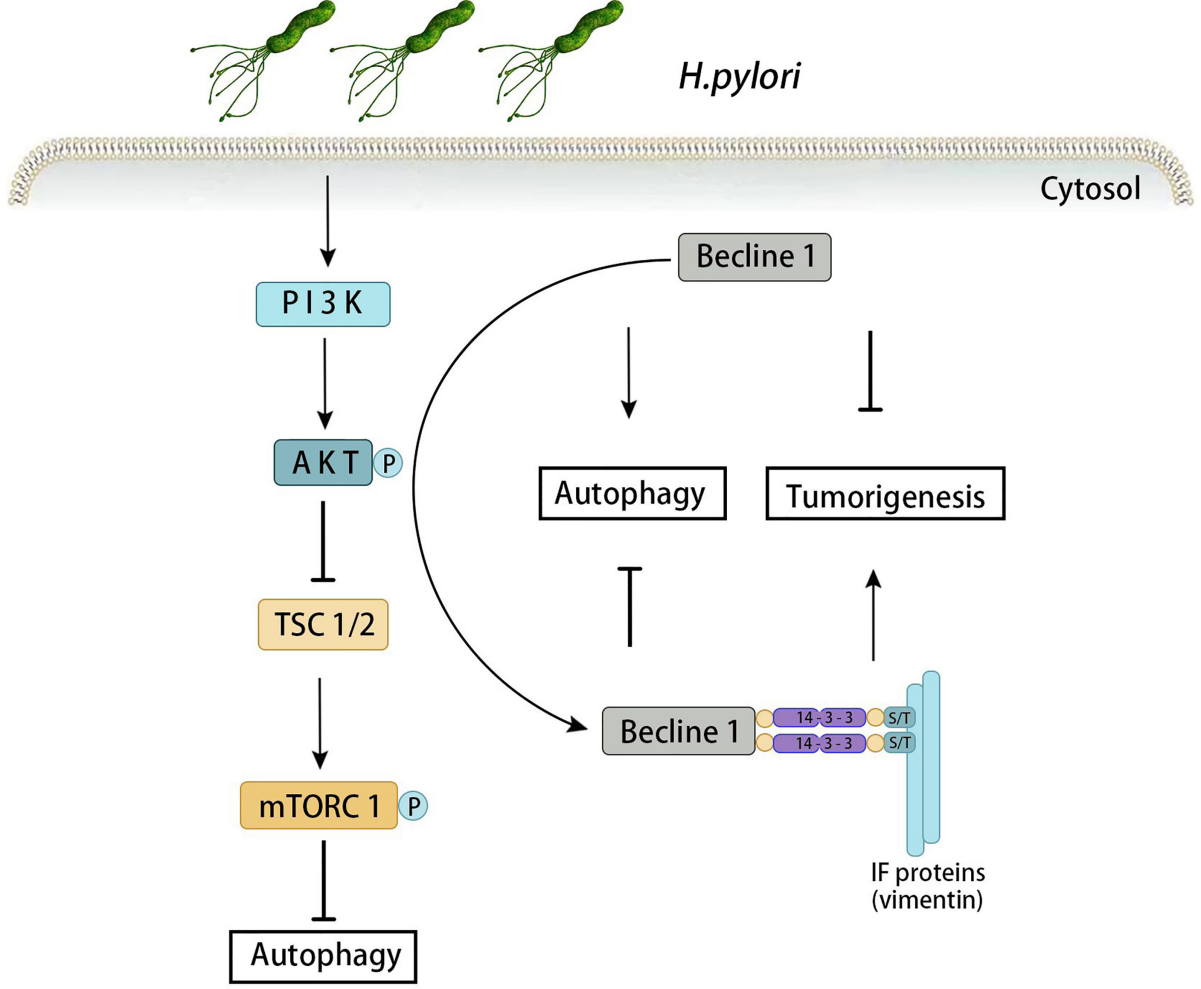
Autophagy induced by PI3K-Akt-mTOR signaling pathway in *H. pylori*-associated gastric cancer. When infected with *H. pylori*, Akt mediates beclin-1 phosphorylation, which activates the autophagy system, increases Vimentin and 14-3-3 protein simultaneously. Increase the expression of Vimentin can induce autophagy, and then promote the Akt-induced carcinogenesis. When Beclin-1 gene is mutated, autophagy can still be activated *via* PI3K-Akt-mTOR if Akt-mediated phosphorylation fails.

The PI3K/Akt/mTOR signaling pathway is an important signal transduction system that can affect the growth, proliferation, survival, metabolism, and angiogenesis of tumor cells. Meanwhile, it especially plays a key role in the invasion, migration and infiltration phases of *H. pylori*-associated gastric cancer (Yang et al., 2017; Ke et al., 2018). PI3K is a lipid kinase family composed of a regulatory subunit (p85) and a catalytic subunit (p110). PI3K promotes phosphorylation of the 3-hydroxyl group of phosphoinositide, and PI3P synthesized by type III kinase acts as the initiation signal to form autophagosome. Akt mediates Beclin-1 phosphorylation and promotes the interaction among the Beclin-1, vimentin and 14-3-3. Increase the expression of Vimentin can induce autophagy, and then promote the Akt-induced carcinogenesis (Wang et al., 2019). When infected with *H. pylori*, Akt mediates beclin-1 phosphorylation, which activates the autophagy system, increases Vimentin and 14-3-3 protein simultaneously. When Beclin-1 gene is mutated, autophagy can still be activated *via* PI3K-Akt-mTOR if Akt-mediated phosphorylation fails. mTOR is an effector molecule downstream of the PI3K-Akt pathway that is an important substance negatively regulating autophagy. By inhibiting autophagy, mTOR regulates cell growth, proliferation and protein synthesis (Kidd et al., 2014). It has been found that the expression of mTOR increased in human and mice gastric epithelial cells when infected by *H. pylori*, and the cause of its increase was largely determined by CagA (Lee et al., 2018). In addition, gastritis was significantly relieved in mTOR knockout (KO) mice which infected with *H. pylori*. These findings support that *H. pylori* promotes gastritis and gastric cancer through the PI3K/Akt/mTOR signaling pathway (**Figure 2**).

### The VEGF Pathway

Vascular endothelial growth factor (VEGF) is a vascular permeability factor that participates in a series of reactions, such as the degradation of the vascular basement membrane, the proliferation of vascular endothelial cells, and the deposition of a new basement membrane. During development, these processes result in embryonic blood vessels being formed, and after birth, they allow existing blood vessels to continue to grow (Feng et al., 2020). Studies have shown that single nucleotide polymorphisms of VEGF are the predictive and prognostic indicators for solid tumors, such as gastric cancer, colorectal cancer, and breast cancer (Gao et al., 2019). The high expression of VEGFR-2 may be related to tumor staging, recurrence and metastasis in gastric cancer. High expression of the VEGFR-2 and the VEGFR-2 rs1870377 A>T gene polymorphisms may a prognostic factor in gastric cancer (Zeng et al., 2019). Beclin-1 overexpression was found to markedly attenuate the ability of gastric cancer cells to migrate and invade possibly due to VEGF hypoexpression (Zhu et al., 2019). It also indicates that VEGF can regulate autophagy related genes and thus affect the occurrence and development of gastric cancer.

### The TGF-β Pathway

TGF-β is an important representative of the TGF-β family, which includes 3 subtypes (β1, β2 and β3). TGF-β1 was first discovered in lymphocytes and monocytes, which is the most common form of TGF-β (Zheng et al., 2020). The TGF-β signaling pathway is connected with the angiogenesis, invasion, metastasis in gastric cancer (Yue and Mulder, 2001).

In terms of autophagy-related research, studies have shown that TGF-β participates in the regulation of autophagy activity in *H. pylori*-associated gastric cancer, which is a powerful activator of autophagy (Liu et al., 2016). In addition, it has been found that the level of TGF-β1 is significantly increased in the *H. pylori*-positive gastric cancer patients (Guo et al., 2012). Meanwhile, the expression of autophagy related genes, BNIP3, Beclin-1, the transformation of LC3 I to II indicated that TGF-β1 enhanced autophagy. To a certain extent, the primary role of TGF-β1 is to inhibit the formation of tumor cells during the early stage of *H. pylori*-associated gastric cancer. However, with the further development of gastric cancer, TGF-β1 cooperates with the VEGF signaling pathway to promote angiogenesis around the tumor, enhancing the ability of tumor cells to invade and metastasize and accelerating the progression of gastric cancer (Wu et al., 2016).

### The miRNA-Related Signaling Pathway

Studies have shown that miRNAs play quite important roles in growth, autophagy and invasion during the pathogenesis of gastric cancer (Tsugawa et al., 2012; Yang et al., 2018). Some microRNA (miRNA)s act as tumor suppressors in gastric cancer, while others act as oncogenic genes by regulating the expression levels of various genes. *H. pylori* is an important factor that can further influence autophagy by regulating the function of miRNAs (Muhammad et al., 2017). The expression of miRNA (miR)-30b are increased most among upregulating miRNAs. Previous literatures have pointed out that *H. pylori* infection can induce changes in miR-30b expression in gastric epithelial cells (Yin et al., 2019). Notably, they found that miR-30b regulates the autophagy process during persistent *H. pylori* infection, thereby promoting the persistence of *H. pylori* infection. Beclin-1 and ATG12, as important proteins involved in autophagy, are new targets of miR-30b (Pang et al., 2020). Besides, miR-30d inhibits the activity of Beclin-1, which prevents further nucleation of autophagy vesicles (Ashrafizadeh et al., 2020). Down-regulating miR-28 can activate the PTEN/PI3K/Akt signaling pathway, which can induce the infiltration and metastasis of gastric cancer (Li et al., 2018). Targeting miR-93-5p can negatively regulate the tumor suppressor AHNAK *via* autophagy and promote the epithelial-mesenchymal transition, and then promote the occurrence and development of gastric cancer (Shen et al., 2020). In addition, methylation silencing caused by *H. pylori* may damage autophagy and promote the occurrence of gastric cancer.

## Drugs Therapy Targeting Autophagy in *H. pylori*-Associated Gastric Cancer

Studies have shown that loss of autophagy may contribute to the development of cancer, but autophagy may also contribute to the development of tumors in turn. On the one hand, autophagy can prevent oxidative stress and DNA damage, which play a key role in inhibiting gastric cancer. On the other hand, autophagy can provide tumor cells with essential metabolites for growth, which can promote the development of gastric cancer (Parzych and Klionsky, 2014; Wang et al., 2021). Changes in the level of autophagy may to a large extent increase the autophagy inducing or inhibiting autophagy activity of tumor cells, and the role of autophagy may be different at different stages of gastric cancer development. In the early stage of tumor growth, tumor vascularization is insufficient and the nutrient supply of cancer cells is limited, thus need enhance autophagy function to provide nutrition for tumors. As soon as the tumor entered the development stage, gene variation accumulated, which inactivated tumor suppressor genes and then reduced autophagy activity (Yang and Klionsky, 2009).Therefore, different drugs play different anti-cancer roles by activating or inhibiting autophagy in different stages of *H. pylori*-associated gastric cancer.

### Chloroquine and Chloroquine Derivatives

Blocking autophagy may be a therapeutic approach to sensitize gastric cancer cells to chemotherapy. Up till now, chloroquine (CQ) and chloroquine derivatives are the lysosomal inhibitors which were most widely drugs used in targeting autophagy therapy. CQ and CQ derivatives have been used in many recent clinical trials and is a most important autophagy modulator in clinical practice. Chloroquine (CQ) blocks autophagy by disrupting lysosome function, and further induces cancer cell death by inhibiting autophagy-mediated ROS20 (Fukuda et al., 2015). Existing literature suggests that, in human cancer cell lines and mouse models, CQ may exhibit significant antitumor activity by inhibiting autophagy induction after cancer treatment (Zhang et al., 2018). In addition, the combination of CQ and cisplatin can improve the sensitivity of cisplatin resistant cells, which indicate that regulation of autophagy can alter the drug resistance of gastric cancer in chemotherapy (Sun et al., 2017).

### Proteasome Inhibitors

Proteasome inhibitors are a novel treatment for cancer, which prevent cancer progression by inhibiting the degradation of ubiquitin-proteasome dependent proteins. Bortezomib is a typical representative of 26S proteasome inhibitors, it has previously been reported primarily for the therapy of multiple myeloma (Richardson and Anderson, 2003). Besides that, research has also found that bortezomib also has anti-tumor effects in other malignancies, such as breast, prostate and colon cancers (Dolcet et al., 2006; Periyasamy-Thandavan et al., 2010; Hong et al., 2012). The increase of autophagy capacity after bortezomib treatment mainly depended on the up-regulation on LC3B by ATF4 (Chen et al., 2011; Befani et al., 2012). In addition, bortezomib was found to inhibit the proliferation in gastric cancer cells, and was more effective in gastric cancer with decreasing the NF-κB activity *via* autophagy (Kao et al., 2014). Bortezomib is a potential novel molecular-targeted autophagy agent with far-reaching promise in the therapy of the unresectable gastric cancer.

### Histone Deacetylase Inhibitors

Histone deacetylase inhibitors have become a class of potential antitumor drugs, which can reactivate genes silenced by histone deacetylation. Histone deacetylase inhibitors can induce the autophagy function through FOXO1 signaling pathway, and can also induce the autophagy function by inhibiting mTOR. In addition, it can induce autophagy through the AMPK-FOXO1 signaling pathway in gastric cancer (Chen et al., 2021). There have been reported that histone deacetylase 2 was increased in human gastric cancer, and histone deacetylase 3 can inhibit the expression of DTWD1, which is a cancer suppressor gene in gastric cancer. Cancer suppressor genes Per1 and Per2 can be promoted by the histone deacetylase inhibitors especially in the human gastric cancer cells (Eckschlager et al., 2017). Sgc-7901 xenograft tumor model showed that Valproic acid (2-propylpentanoic acid, VPA) could inhibit histone deacetylase 1/2(HDAC1/2) activity and induce autophagy in gastric cancer cells, which can further inhibit the HDAC1/PTEN/Akt signaling pathway as well as regulate Bclin-1 and Beclin-2 (Sun et al., 2020).

### IFN-γ

The natural killer cell (NK) cells are cytotoxic innate lymphocytes that are critical to anti-infection response and play an important role in resisting viral infection, they also have the unique ability to kill cancer cells, limit tumor growth and metastasis (Zingoni et al., 2017). Recent research has confirmed the significance of NK cells in promoting the immune response especially in tumors which intrinsic autophagy loss. In fact, inhibiting autophagy genes lead the massive NK cells proliferation, which can suppress the progression in solid tumors. NK cell activity is decreased in gastric cancer, and gastric cancer mesenchymal stem cells promote tumor growth and inhibit NK cell function through the mTOR signaling pathway. Interferon -γ (IFN-γ) is an important mediator of innate and adaptive immunity, which also an important tumor suppressor (Billiau et al., 1998; Yang et al., 2022). IFN-γ induces a variety of immunomodulatory molecules by regulating the expression levels of multiple genes, such as promote NK cell activity^119.^IFN-γ induces gastric epithelial autophagy by increasing Beclin-1, which inhibits carcinogenesis in the gastric mucosa *via* further reducing epithelial cell apoptosis (Tu et al., 2011). In conclusion, as a means of surviving in limited nutritional conditions, restore and replace dysfunctional NK cells may be an attractive therapeutic strategy. Targeting autophagy to activate NK cells activity has great potential in the treatment of gastric cancer patients (Zhou et al., 2018).

### Metformin

Metformin is an oral drug, commonly used to treat type 2 diabetes. With more and more understanding of metformin, the function of metformin can improve the prognosis of cancer patients has been identified, thus the randomized controlled trials were implemented to verify the capacity of metformin to prevent cancer or improve cancer outcomes (Kasznicki et al., 2014). Furthermore, some reports suggest that the potential of metformin anticancer properties are related to the regulation of many miRNAs. For instance, metformin targets miRNA-222 to inhibit lung cancer multiplication, and metformin targets miRNA-21 and miRNA-145 to treat relapsed colon cancer (Quinn et al., 2013). Recent studies have shown that metformin acts as an autophagy agonist that induces autophagy by activating AMPK, and this survival promoting process can inhibit gastric cancer growth, reduce invasion and migration of gastric cancer cells, and inhibit the carcinogenic properties of gastric cancer stem cells (Ben Sahra et al., 2011; Meng et al., 2015; Lee et al., 2020). In addition, studies have further suggested that metformin promotes Beclin1-dependent autophagy to inhibit the progression of gastric cancer, which associated with changing the mRNA expression levels of LC3B and Beclin-1 (Takahashi et al., 2014).

### Calcium Ion Channel Inhibitors

The involvement of Calcium ion (Ca2+) channels in the regulation of autophagy as an autophagy trigger was discovered in the early 1990s. Studies suggest that intracellular Ca2+ signaling is related to autophagy, the increase of Ca2+ in free cells can activate AMPK and trigger autophagy. In addition, intracellular Ca2+ signaling is required for mTOR dependent autophagy and the lysosomal Ca2+ signaling pathway activates autophagy through the calcineurin. Ca2+ signaling is enhanced when cells are exposed to stress, that is, the induction of autophagy requires an increase in Ca2+ levels (Kondratskyi et al., 2013). Nifedipine and other calcium ion channel inhibitors, may be the potential candidates for targeted autophagy in the therapy of gastric cancer (Filippi-Chiela et al., 2016).

### Statins

Recent reports suggest that statins modulate autophagy in tumor cells, and statin contribute to decrease cholesterol levels in the membrane of the phagosome, which in turn activates the host-induced autophagy (Zhu et al., 2021). The nuclear factor-κB is related to the autophagy induced by statins, which can also induce autophagy activated by AMPK, leading to the endoplasmic reticular cell response (Li et al., 2021). In addition, statins can reduce the risk of heterochronous gastric cancer in patients without *H. pylori* infection (Liao et al., 2016). These results suggest that statins can not only reduce infection caused by *H. pylori*, but can play a significant role in the therapy of gastric cancer by targeting the activation of autophagy pathway.

### Astaxanthin

Astaxanthin, a carotene pigment, is a beneficial compound with excellent antioxidant effects (Hussein et al., 2006). It can inhibit lipid peroxidation, protects cells and reduce DNA damage, enabling proteins inside cells to function better. Clinically, astaxanthin reduces the incidence of atherosclerosis by prolonging the oxidation time of low-density lipoprotein (LDL) and inhibits apoptosis by reducing reactive oxygen species (ROS) (Ikeda et al., 2008; Kim et al., 2018). A recent report suggests that astaxanthin protects AGS cells from *H. pylori* infection by upregulating cellular protective autophagy and modulating AMPK pathways (Lee et al., 2020). In this study, astaxanthin pretreatment of corresponding cells resulted in increased protein expression levels of AMPK phosphorylation, as well as increased fluorescence of LC3B and acidic vesicle organelles in immunofluorescence, which suggest that astaxanthin can induce autophagy. Further experiments showed that astaxanthin induced autophagy by increasing AMPK phosphorylation and inhibiting its downstream target mTOR. Because mTOR inhibits ULK1 phosphorylation, astaxanthin activates ULK1 and then induces autophagosome formation by influencing the interaction between P62 and LC3B-II in cells. Therefore, astaxanthin has important prospects in targeting autophagy therapy in gastric cancer.

### The Combination of Catechins and Sialic Acid

Catechin is a substance with multi-hydroxyl structure, which can play a variety of pharmacological effects, such as antioxidant, antibacterial and anti-tumor (Mabe et al., 1999). Sialic acid, a tetrapeptide containing arginine, is found naturally in saliva and has a powerful antioxidant function (Chun et al., 2007). The potential of catechins and sialic acid for the prevention and therapy of *H. pylori* infected gastric epithelial cells has been explored *in vitro* and *in vivo* (Yang et al., 2008; Yang and Chien, 2009; Yang et al., 2013). The combination of catechins and sialic acid can significantly enhanced autophagy mediated by Beclin-1. Catechins and sialic acid reduce *H. pylori* density on the surface of the gastric lumen through their antibacterial and anti-adhesion properties firstly, then they inhibit the production of ROS derived from CD68 and NADPH oxidase, which can induce autophagy mediated by Beclin-1, reduce caspase-1 activation and IL-1β secretion subsequently. In addition, the combination of catechins and sialic acid can eradicate *H. pylori* in a dose-dependent manner, which can be the novel anti-*H. pylori* drugs and have important significance in the therapy of *H. pylori*-associated gastric cancer.

## Discussion

The mechanisms of autophagy regulated by *H. pylori* infection in gastric epithelial cell are quite complicated. The signaling pathways of gastric cancer that are related to *H. pylori* infection mediated by autophagy involve a multichannel and multifactor protein network regulation system. Various signaling pathways play their respective roles and can interact with each other. Once a signaling pathway is activated or abnormally induced, it may cause a series of cellular immune damage, eventually leading to the occurrence of gastric cancer. Therefore, targeted autophagy therapy of *H. pylori*-associated gastric cancer has become a new hot spot, representing a fresh orientation for the therapy of gastric cancer that combines different signal transduction pathways. Through targeted autophagy therapy, seeking multiple regulatory points of autophagy pathways and blocking signal transduction, thus finally achieve inhibition of gastric cancer development.

## Author Contributions

YY wrote the manuscript; XS and CX revised the review. All authors contributed to the article and approved the submitted version.

## Funding

The National Natural Science Foundation of China (No. 81060038 and 81270479), grants from the Jiangxi Province Talent 555 Project, and the National Science and Technology Major Projects for “Major New Drugs Innovation and Development” of China (No. 2011ZX09302-007-03).

## Conflict of Interest

The authors declare that the research was conducted in the absence of any commercial or financial relationships that could be construed as a potential conflict of interest.

## Publisher’s Note

All claims expressed in this article are solely those of the authors and do not necessarily represent those of their affiliated organizations, or those of the publisher, the editors and the reviewers. Any product that may be evaluated in this article, or claim that may be made by its manufacturer, is not guaranteed or endorsed by the publisher.
